# Helpful and hindering factors for remission in dysthymia and panic disorder at 9-year follow-up: A mixed methods study

**DOI:** 10.1186/1471-244X-8-52

**Published:** 2008-07-01

**Authors:** Cecilia Svanborg, Sofie Bäärnhielm, Anna Åberg Wistedt, Kim Lützen

**Affiliations:** 1 Department of Clinical Neuroscience, Psychiatry Section, Karolinska Institute, Psykoterapienheten City, Karlavägen 53, 11449 Stockholm, Sweden; 2 Transcultural Centre, Stockholm County Council, S:t Göran's Hospital, Stockholm, Sweden; 3 Department of Clinical Neuroscience, Psychiatry Section, Karolinska Institute, S:t Göran's Hospital, Stockholm, Sweden; 4 Department of Clinical Neuroscience, Psychiatry Section, Karolinska Institute, Huddinge University Hospital, Huddinge, Sweden

## Abstract

**Background:**

A better understanding is needed of factors behind the long-term outcome of dysthymic and panic disorders. Combining patients' perceptions of factors that help and hind remission with objective assessments of outcome may give greater insight into mechanisms for maintaining recovery.

**Methods:**

Twenty-three dysthymic and 15 panic disorder patients participated in a 9-year follow-up investigation of a naturalistic study with psychotherapy and antidepressants. Degree of remission was determined by reassessments with SCID-I & II interviews, self-reported symptoms and life-charting (aided by case records). Qualitative content analysis of in-depth interviews with all 38 patients was done to examine the phenomenon of enduring remission by exploring: 1) perceived helpful and hindering factors, 2) factors common to and specific for the diagnostic groups, 3) convergence between patients' subjective views on remission with objective diagnostic assessments.

**Results:**

About 50% of the patients were in full or partial remission. Subjective and objective views on degree of remission generally converged, and remission was perceived as receiving 'Tools to handle life'. Common helpful factors were self-understanding, enhanced flexibility of thinking, and antidepressant medication, as well as confidence in the therapist and social support. The perceived main obstacle was difficulty in negotiating treatments. Remitted had overcome the obstacles, whereas many non-remitted had problems expressing their needs. Patients with dysthymia and panic disorder described specific helpful relationships with the therapist: 'As a parent' versus 'As a coach', and specific central areas for change: self-acceptance and resolution of relational problems versus awareness and handling of feelings.

**Conclusion:**

A general model for recovery from dysthymic and panic disorders is proposed, involving: 1) understanding self and illness mechanisms, 2) enhanced flexibility of thinking, and 3) change from avoidance coping to approach coping; and recognising that a vehicle for this change is a helpful relationship to the health care provider. The perceived needs of specific treatment ingredients suggest that it is essential to differentiate between early-onset dysthymia and secondary depressions. The perceived access problems will be further investigated.

## Background

Despite repeated treatments, many patients with dysthymia and panic disorder do not achieve remission or have persistent residual symptoms and functional impairments that pose a risk of relapse and recurrence [1-3]. A general conclusion is that the effectiveness of existing treatments is limited with regard to long-term remission. Thus, a better understanding is needed of helpful treatment ingredients and hindering factors, as well as of the phenomenon of enduring remission.

Comorbidity, especially with personality disorders, has been shown to be a factor that renders remission more difficult for patients with dysthymic and panic disorders [4,5]. Both disorders are characterized by high frequencies of comorbid avoidant and obsessive-compulsive personality disorders [6]. Concerning the influence of comorbid depression on the outcome of panic disorder the findings are somewhat inconsistent [7,8]. We suggest that a confounding factor may be insufficient differentiation between primary, early-onset dysthymia and secondary depressions. It is reasonable to assume that when panic disorder is successfully treated, secondary depressions may recede but that patients with primary dysthymic disorder may need other specific treatment ingredients. In order to explore factors for maintaining remission, we wanted to compare patients with dysthymia and patients with panic disorder without dysthymia.

Psychotherapy has long been promoted as providing enduring change. An important question, however, is how to understand the possible mechanisms involved in such a desirable outcome. Another long-standing issue is whether the benefits of psychotherapy derive from features common to all psychotherapies – such as the healing setting, psycho-education, and the therapeutic relationship – rather than to specific factors related to the type of therapy [9]. A neglected circumstance is that the complex interactions between common and specific factors are associated with the character of a patient's problems. Bohart [10] proposed an alternative model where patients' self-healing capacities are the common factor which makes therapy work. According to this model, patients take whatever techniques are provided and use them to obtain the needed changes. Moreover, they may have fairly elaborate perceptions of the obstacles to change. From the perspective of the patient as the expert on what works, patients' opinions about helpful and hindering factors for remission ought to promote the understanding of factors that affect outcome. Furthermore, an investigation of patients with dysthymia and panic disorder might show whether the perceived factors are similar or dissimilar.

In a comprehensive review, Elliott et al. [11] summarised research on clients' experiences of psychotherapy under five helpful categories: (a) facilitative therapist characteristics, (b) client self-expression permitted, (c) experiencing supportive relationship, (d) client self-understanding, and (e) the therapist encouraging clients to practise new skills outside of therapy. To our knowledge, however, no studies have reported the perceptions of patients with a long duration of illness, trying to relate the findings to different diagnoses and degree of remission at long-term follow-up.

The purpose of the present study was to examine the phenomenon of remission by investigating the perceptions of patients with dysthymic and panic disorders with different long-term outcomes at 9-year follow-up. Specifically, we wanted to explore: (1) Perceived helpful and hindering factors, (2) Common and specific factors for the diagnostic groups, and (3) Convergence between patients' subjective views on remission with objective diagnostic assessments.

## Methods

### Design

We used a combination of quantitative and qualitative methods, conceptualizing the study from a pragmatic theoretical paradigm [12]. The quantitative and qualitative data were collected at the same time; the sampling was purposeful in accordance with qualitative method (selection of information-rich cases) [13]; long-term outcome was evaluated with diagnostic assessments, evaluation of the illness course and quantitative measures; and in-depth interviews aimed at understanding the perceptions of the target population. Informed consent was obtained and the Ethic Committee of the Karolinska Institute approved the study.

### Participants

Participants were selected from two previous naturalistic treatment studies (conducted 1992–1996) within psychiatric care in Stockholm, Sweden, if: (a) the primary Axis I diagnosis was either dysthymia or panic disorder, (b) illness duration was longer than 2 years when included in the original studies, and (c) not both dysthymia and panic disorder. In 2004, 83 patients were asked to participate in the long-term follow-up; 42 (51%) agreed to participate, of whom 38 fulfilled diagnostic criteria at follow-up. There were no differences between participants and non-participants regarding age, sex, Axis I diagnosis, frequency of comorbid Axis II diagnosis, symptom level at baseline and after 2 years. The sample was therefore assessed as being representative of all 83 patients and is described in a previous study [14].

Participants were 23 patients with dysthymia (aged 34–68, median 44 years), mainly with early onset, and 15 patients with panic disorder (aged 32–51, median 40 years), mainly with agoraphobia. The characteristics of participants at follow-up are shown in Table 1. Almost all participants had received both psychotherapy (mainly individual psychodynamic and/or cognitive-behavioural) and antidepressant medication during their treatment histories. Five participants had only had psychotherapy (4 with dysthymia, 1 with panic disorder) and 2 participants had only had antidepressant medication (1 with dysthymia and 1 with panic disorder).

**Table 1:** Degree of remission in 38 patients with panic disorder and dysthymia at a 9-year follow-up examination

Characteristics	Panic disorder			Dysthymia		
		
	Remission (n = 3)	Partial remission (n = 4)	Non-remission (n = 8)	Remission (n = 6)	Partial remission (n = 7)	Non remission (n = 10)
Women, n (%)	1 (33)	2 (50)	7 (88)	3 (50)	4 (57)	6 (60)
Working full time, n (%)	2 (66)*	4 (100)	4 (50)	6 (100)	7 (100)	5 (50)
Married/cohabitant n (%)	2 (66)	2 (50)	5 (63)	5 (83)	4 (57)	1 (10)
Personality disorder, n (%)	0	0	3 (38)	0	2 (29)	10 (100)
Current medication, n (%)	0	1 (25)	4 (50)	1 (17)	2 (29)	4 (40)
Current psychotherapy, n (%)	0	0	0	0	2 (29)	2 (20)
GSI **, m (range)	.28 (.23–.32)	.58 (.06–.92)	1.41 (.45–2.7)	.21 (.00–.34)	.84 (.47–1.7)	1.49 (.38–2.4)

*One unemployed

** GSI = Global Severity Index= mean SCL-90 score

### Assessment of long-term outcome

Participants were reassessed by the first author with *SCID-I & II interviews *[15,16] and self-reported psychiatric symptoms with the *Symptom Checklist-90 (SCL-90/BSI) *[17]. There is strong evidence for using the mean total score of the SCL, the Global Severity Index (GSI), as an expression of overall neurotic illness [18]. The diagnostic assessment was followed by a *semi-structured, retrospective Life-charting interview developed from the NIMH Life Chart Methodology (NIMH-LCM)*, investigating course, treatment (aided by case records) and significant life events from the first day of remembrance until the day of the interview [19]. An investigation of the stability of change compared to the 2-year outcome and the influence of comorbid personality disorder (PD) is reported in a previous article [14]. In summary, the analysis showed that comorbid PD at baseline was a negative prognostic factor irrespective of Axis I diagnosis and that participants with panic disorder had a less stable outcome.

#### Definitions of outcome

The time interval used to define outcome was six months prior to follow-up, according to a commonly used definition of recovery [20,21]. *Remission and partial remission*: Participants did not meet DSM-IV criteria for dysthymia or panic disorder, the former having no or minimum symptoms and no functional impairment, the latter having some symptoms or functional impairment. *Non-remission*: Participants were fulfilling DSM-IV criteria for dysthymia or panic disorder.

### Narrative data collection

The first author, a senior psychiatrist and a licensed psychotherapist in cognitive behavioural psychotherapy, conducted all the interviews. The narrative data were collected after the life-charting interview. The detailed information about treatments over time helped participants to remember and reflect and the interviewer to link statements to specific treatments.

The narrative data were collected by open-ended interviews, lasting about 30 minutes and focused on the following questions: (1) How are you today? Are you recovered? (2) What governed which and how long treatment you received? (3) What has been helpful? (4) Have you changed? In what way? How do you explain this change? Have you learnt something? (5) What has maintained your troubles? (6) Are there any external circumstances that have helped or hindered? (7) Do you have any experiences from health care that have been a hindrance? (8) How do you view your future?

Follow-up questions were used to clarify ambiguities and elaborate responses with consideration for the participants' concerns. All interviews were digitally recorded and transcribed verbatim.

### Qualitative data analysis

Narrative data were analysed with qualitative content analysis, which "refers to a qualitative data reduction and sense-making effort that takes a volume of qualitative material and attempts to identify core consistencies and meanings [22]." Epistemologically, the pieces of data were seen as both informational units and constructions of facts to be interpreted [23]. The first author (CS) formed a primary team together with the second author (SB); the fourth author (KL) was an external auditor. SB is a psychiatrist and KL is a professor in Caring Science, both with expertise in qualitative methods. The analysis was conducted as follows: (1) All interviews were read open-mindedly in order to gain an overall impression. (2) The text was reread several times and the meaning units were given an open code close to the wordings of the participants. (3) The meaning units were coded "bottom-up" from subcategories into categories for each subject. (4) The codes and categories, arrived at independently, were discussed by CS and SB. Agreement was good and differed mainly in the choice of words. In the event of disagreement, CS was given the preferential right of interpretation on account of her direct encounters with the participants. (5) CS reviewed all subcategories and categories for all cases with the aid of the computer software NVivo 2.0 [24], comparing them with each other to identify commonalities and redundancies. (6) In the subsequent comparative cross-analysis, the category system was further analysed for convergence and divergence into a coherent picture. (7) The frequency of participants for each category was noted: *General*, applied to all or all but 1 case; *typical*, applied to more than half up to the cut-off for general; *variant*, applied to several cases up to cut-off for typical; *rare*, applied to 1–2 cases which were not included in the results or tables [25]. (8) KL made an external audit of the category system and the transcripts with noted categories and subcategories. Agreement with the outcome analysis was satisfactory.

## Results

### Long-term outcome

At the follow-up, 26% (n = 6) of patients with dysthymia and 20% (n = 3) of patients with panic disorder were in remission according to SCID-I-interviews and symptom measures. According to the life-charting, they had been in remission between 1–8 years (median 4 years). Including partial remission individuals, 57% (n = 13) of patients with dysthymia and 47% (n = 7) of patients with panic disorder had improved. Both diagnostic groups had predominantly maladaptive traits in cluster C according to DSM-IV (mainly avoidant, obsessive-compulsive and dependent), (dysthymia 57%, n = 13, panic disorder 80%, n = 12), but traits in cluster A (mainly paranoid) and cluster B (mainly borderline) were also prevalent.

The subjective perceptions about life today, change and the future were generally convergent with diagnostic assessments according to DSM-IV definitions. General for remitted participants were perceptions of having received 'Tools to handle life', e.g.: "*it is positive, now I have the requirements to handle my life*", "*the future is bright, as I have received the tools to push my life in the wanted direction*."

### Common factors

The analysis of the narrative data concerning perceived help and hindrance resulted in both common and specific categories for the diagnostic groups, as shown in Tables 2 and 3. The text describes variations between groups with different outcomes and diagnoses, illustrated with some short quotations and longer excerpts for central categories.

**Table 2:** Common categories of perceived helpful and hindering factors for remission based on a 9-year follow-up examination of 38 patients with panic disorder and dysthymia

**Helpful factors**	**Hindering factors**
Successful negotiations	Difficult negotiations
- Fought for my request	- Misunderstood and rejected
- Chose my therapist	- The patient is the underdog
- Found financing for psychotherapy	- Problems financing psychotherapy
- Enough time	- Too little time
Antidepressant medications	Medication problems
- Stabilizes	- Fear
	- Side effects
	- No problem solving
Allowed to express myself	Therapist too non-directive
Confidence in the therapist	Lack of confidence in the therapist
Understanding myself and mechanisms	Lack of understanding
Reasoning with myself	Reasoning with myself is not enough
Important relations to others*	Unresolved relational problems*

* = Non-treatment factors

**Table 3:** Specific categories of perceived helpful and hindering factors for remission based on a 9-year follow-up examination of 38 patients with panic disorder and dysthymia

**Panic disorder**		**Dysthymia**	
	
**Helpful factors**	**Hindering factors**	**Helpful factors**	**Hindering factors**
Therapist as coach	Phobias*	Therapist as parent	Mistrust of others*
Relaxation techniques	Fear of anxiety*	Experiential and creative techniques	Sensitive to confirmation*
Awareness and handling of feelings	Difficult handling bodily sensations and feelings*	Self-acceptance and compassion	Blaming self or others*
Exposure gave confidence		Feedback from others in group	Difficulties in close relations*
		Resolved relational problems	
		Several therapies important	

* = Non-treatment factors

The most common, almost general category was 'Difficult negotiations', defined as problems with access to treatment. Almost all participants described the process of receiving psychotherapy as a struggle about financing and time. Other hindering factors were perceptions of being misunderstood, rejected and powerless due to problems in expressing their needs forcefully enough. This was typical for those not in remission but also variant for others, especially early in the treatment history, e.g.: *"I had no choice," "it's bad when they reject someone who is already an underdog," "she ruled by saying that there were no alternatives."* In contrast, the category 'Successful negotiations,' defined as overcoming access problems, was typical for participants in full or partial remission. Faced with problems about the frames of treatment, they had fought and found solutions, e.g.: *"I'm stubborn, I made contact again", "I know what I want and do not want," "the first therapist was odd, I wanted another one."*

'Antidepressant medication stabilizes' was a typical common helpful factor, e.g.: "*gave me stability," "made such a difference."* However, 'Medication problems' was a typical hindering factor with complaints about physical and/or mental side effects resulting in dropout. Variant perceptions were that medication did not solve the basic problems or fear of medication. 'Confidence in the therapist' and 'Lack of confidence' were typical for both diagnostic groups. The only category that could be linked to a specific psychotherapy was 'Therapist too non-directive', associated with psychodynamic psychotherapy, e.g.; "*therapist was silent, I didn't know what to say or do*." 'Understanding myself and mechanisms' was the most common helpful factor, typical for all participants. For those in remission, it was a general category and described in terms of a tool to handle distress, e.g.: "*it's just that I know what it is, that makes it possible for me to handle it*" (original emphasis underlined). Understanding was linked to experiences in psychotherapy or reading books and magazines about mental health. 'Lack of understanding' was a variant hindering factor, which had made negotiations about treatments difficult.

'Reasoning with myself', defined as an enhanced ability to reflect on and modify own thoughts, was a typical helpful factor, irrespective of type of psychotherapy, e.g.: "*I think that I have received the tools to reverse things, by being aware about how I think it is easier to reverse a depressed state*." 'Important relations to others' (support from partner and friends, becoming a parent) was a typical and highly valued helpful factor irrespective of outcome. 'Unresolved relational problems' was a typical hindering factor for those in partial remission or non-remission, with predominance of participants with dysthymia.

### Specific factors for panic disorder

Participants with panic disorder and dysthymia described specific helpful relationships to the therapist, irrespective of type of psychotherapy or outcome. In panic disorder it was typically described as good collaboration ('Therapist as coach'), e.g.: "*like a coach," "educated me," "helped me reach my goals."* 'Exposure gave confidence' and 'Relaxation techniques' were variant helpful factors. Relaxation was described as a help to control or tolerate sensations; the former by non-remitted participants, the latter described as follows by a participant in remission: *"all this together gets you to relax and not to be so afraid of external impressions and to come to grips with your emotional turmoil."* A general helpful factor for those in remission was 'Awareness and handling of feelings', illustrated by this excerpt:

"But first and foremost, for the first time in my life I have learnt to notice what I feel and, yes, reflect a little about how to handle it. So I have a strategy for times when it's difficult. I've never had that before, it used to become panic directly. Yes, I have received tools to hold on to in times of storm and thunder."

The enhanced capacity to identify, tolerate and handle feelings, especially anger, was perceived as a tool for "*putting my foot down in a way I didn't do before*," thereby reducing overload and stress. 'Difficult handling bodily sensations and feelings', 'Fear of anxiety' and 'Phobias' were typical hindering factors for those not in remission. Partial remission individuals had hindering persistent phobias.

### Specific factors for dysthymia

In dysthymia, the relationship with the therapist was typically described as a caring relation ('Therapist as parent'), e.g.: "*cared for me;" "secure;" "loving and accepting;" "made me feel liked;" "set limits." *'Experiential and creative techniques' and 'Feedback from others in group' were variant helpful factors, irrespective of outcome. General helpful factors for those in remission were 'Self-acceptance and compassion', 'Resolved relational problems' and 'Several therapies important'. The experience of a caring relationship with the therapist was an important factor in gaining a better self-acceptance for those in remission. The following quotation from a woman in remission from dysthymia illustrates this:

"Yes, it was just that I was met by opposition and received new perspectives on life or tools that, you had not those blinkers against positive things, so instead I have started to be able to receive good things that others do for me too and grow with that. I grew through the support I received ...()...Yes, I think that I have received loving care, without those demands my parents had on me, and I have been allowed to grow through communication."

The hindering factors 'Mistrust of others' and 'Blaming self and others' were typical for individuals in partial and non-remission. 'Difficulties in close relations' was a general factor, and 'Sensitive to confirmation' was a typical factor for non-remission.

## Discussion

### Common helpful and hindering factors

At the 9-year follow-up, the majority had not achieved full remission and a previous analysis had shown a negative impact of comorbid personality disorder and that patients with panic disorder had a less stable outcome [14]. These findings are in accordance with other naturalistic long-term follow-up investigations showing high rates of persistent illness and recurrences in dysthymia and panic disorder with agoraphobia [2,3,8]. In the present study, we sought to expand these findings by combining objective assessments with qualitative methods to get a better understanding of helpful and hindering factors for remission. Furthermore, we wanted to explore whether there are any common and specific factors for patients with dysthymic and panic disorders.

Participants in remission perceived that self-understanding, learning to reason with oneself and social support as well as specific changes for the diagnostic groups had built a sense of empowerment and optimism about the future. Repeatedly they described the gains as 'Tools to handle life', which is congruent with the suggestion that environmental mastering and self-confidence are central domains for recovery and psychological well-being [26,27]. The common helpful factors in psychotherapy are consistent with previous research on patients' experiences [11]. In the present study, recovered participants described that they had achieved an understanding of illness that facilitated a behavioural change from avoidance to approach coping. A new, interesting finding is that participants valued enhanced flexibility of thinking ('reasoning with myself'), irrespective of the psychotherapeutic approach. Enhanced flexibility of thinking and capacity to generate alternative perspectives can be connected to different concepts and types of psychotherapy, e.g. 'metacognition' and 'decentration' for cognitive-behavioural prevention of depression relapse [28,29], and development of 'reflective functioning' in psychoanalytically informed treatment of borderline personality disorder [30]. A mechanism for relapse prevention may be that when patients have a more flexible relation to the content of their thoughts, this reduces the need for avoidant cognitive processing. The significance of avoidance for persistence of illness is supported by other findings, e.g. the links between avoidance coping and stress generation [31], and avoidance of distressing autobiographical memories as a vulnerability factor in depression [32].

The perception that antidepressant medication helped is expected, as are the problems with this medication. For example, it has been suggested that antidepressants have an effect on the common factor of negative emotionality (neuroticism) in depressive and anxiety disorders [33]. Concerning contextual factors, many participants stressed the positive impact of social support and meaningful relations. Perceived social support has been shown to predict long-term outcome in different types of psychiatric illness, e.g. alcohol use disorders [34], major depressive disorder [35] and bipolar disorder [36].

The main perceived obstacle to remission concerned the frames of treatment, mainly expressed as a struggle to receive and choose psychotherapy. Patients in full or partial remission had overcome these barriers, e.g. by contacting the 'ombudsman' for patients, negotiating for longer courses of psychotherapy and an approach-oriented coping when there were strains in the relation to the health-care provider. In contrast, participants with greater disabilities had more difficulties in getting access to adequate treatment. They had problems with articulating their needs, leading to feelings of being misunderstood and rejected. Their perceptions could be an effect of their current states; however, the experiences were validated in the case records. Thus, the findings in the present study, together with previous analysis of the sample, suggest that low capacity to negotiate and adhere to treatments is one factor that makes comorbid personality disorder a negative prognostic factor in long-term, naturalistic studies.

### Specific alliance factors

The finding that a good relationship with the therapist and the physician was a highly valued helpful factor is expected. The link between the alliance and outcome is well established and is often seen as a common factor across therapeutic disciplines [37,38]. However, it has been questioned whether alliance facilitates other factors or is curative in itself [39]. The instruments measuring alliance share two elements called "personal attachments or bonds" and "collaboration or willingness to invest in the therapy process" [40]. These elements might have specific significance for patients with dysthymia and panic disorder as the participants in the diagnostic groups surprisingly described the relationship with the therapist differently. The fact that dysthymic participants described their relationship with therapists in terms of personal attachment rather than collaboration generates the hypothesis that they had a greater need of attachment than participants with panic disorder. Patients with panic disorder might benefit especially from collaboration with therapists who can teach them concrete principles of exposure and cognitive modifications that counteract beliefs of vulnerability, whereas those with dysthymia might benefit more broadly from interventions that target their unmet needs for attachment, pleasure and self-esteem.

### Specific factors for panic disorder

For panic disorder, there was a striking difference between participants with different outcomes regarding awareness, tolerance and dealing with feelings. Recently, systematic relearning of safety in response to both internal and external phobic cues, with particular emphasis on altering responses to emotional arousal, has been highlighted as the central element of efficacious treatment for panic disorder [41]. Furthermore, research suggests that treatments that incorporate affect control strategies, e.g. relaxation training, distraction and benzodiazepine use, are less efficacious than exposure-based procedures over time [41]. Our findings are consistent with this body of research, indicating that elements of treatment can be used as 'safety seeking behaviours' that reduce efficacy of exposure and maintain the disorder by preventing disconfirmatory experiences concerning fears of bodily sensations [42]. Remitted participants had perceived that emotional awareness and management had built a platform for more adaptive stress coping strategies than avoidance and control. These behavioural strategies are closely linked to the major features of avoidant and obsessive-compulsive personality disorders, implying that a mechanism for enduring remission might be that patients learn more flexible strategies concerning emotional experiences. A mechanism for recurrence might be that treatments which help patients with panic disorder avoid or control sensations are ineffective in the long run.

### Specific factors for dysthymia

General helpful factors for those in remission from dysthymia were to learn self-acceptance and to resolve relational problems. Explicitly, participants not in remission brought up unresolved relational problems, blaming and mistrust as hindrances. Two empirically supported psychotherapies are designed for the treatment of chronic depression and dysthymia, i.e. interpersonal psychotherapy (IPT) and cognitive behavioural analysis system of psychotherapy (CBASP) [43,44]. Both psychotherapies emphasize the importance of learning to resolve interpersonal problems as crucial for overcoming chronic depression. The participants in the present study validate this focus. However, all except one of the participants with persistent dysthymia had experience of psychotherapy, and the majority had none or inadequate antidepressant medication according to case records and life-charting. This raises several questions for the recognition and treatment of patients with dysthymia in clinical practice as antidepressant medication is an evidence-based treatment [45]. The present study indicates that patients with dysthymia and comorbid personality disorder are less likely to receive adequate treatment and that better cooperation between the clinician and the psychotherapist is warranted.

### Methodological aspects

An important strength is that we used a combination of quantitative and qualitative methods in order to examine the phenomenon of remission and the perceived reasons for positive and negative outcome [46]. The sample was assessed to be representative for dysthymic and panic disorder patients with long illness duration in psychiatric care. Participants were recorded in detail regarding clinical characteristics and treatments. The qualitative analysis provided a coherent framework for understanding long-term remission and generated hypotheses that can be tested in future studies. Other strengths are the long follow-up period and two groups of patients with long experience of psychiatric care who have been able to describe their perceptions and illness course with life-charting.

However, there are some limitations. Comorbidity between dysthymia and panic disorder is very common. We wanted to study cases without this kind of comorbidity in order to delineate possible specific factors. This is an exploratory study where a limited number of participants had experienced various treatments and our interpretations of the data should be considered within the context of this method. Participants were asked about their perceptions of treatment over their lifetime. This implies a risk for memory bias and it has to be emphasised that the participants had different degrees of awareness and communication ability. However, the life-charting procedure and rich access to case records diminished these limitations. The first author is a cognitive behavioural therapist and psychiatrist, and this has influenced the analytical work. We acknowledge that the analytic process is grounded in subjectivity. While recognising that the analysis is a co-construction of the participants' reality, we strived to minimize researcher bias [47]. We handled the limitation by having two primary coders, "bracketing" our assumptions during the analysis and coding "bottom-up" into categories [48]. Other measures to ensure trustworthiness of the study were careful assessments and descriptions of the sample, the use of an additional "auditor", and grounding the results with examples. Despite the above-mentioned limitations, coherence of the framework, along with good correspondence with results from other studies of theoretical relevance, suggest that the results contain validity.

## Conclusion

Based on the findings from this study and other research, we propose a general model for enduring remission from dysthymic and panic disorders that involves functional changes, i.e. (1) understanding self and illness mechanisms, (2) enhanced flexibility of thinking, and (3) change from avoidance to approach coping. A necessary vehicle for this change is a helpful relationship with the therapist and the clinician. In addition, differentiation between early-onset dysthymia and secondary depression is essential since patients with dysthymia and panic disorder seem to need change in specific areas. Concerning panic patients, clinicians need to recognise that elements of treatment can function as safety seeking behaviours. A key target in the treatment of patients with panic disorder with agoraphobia might be training of emotional awareness, tolerance and management. In treatment of patients with early-onset dysthymia, the need for an alliance factor of personal attachment to gain self-acceptance will be further investigated. Moreover, this study indicates that patients with personality disorders have difficulty in negotiating treatments, which may be a factor that contributes to a persistent course. We will deepen the investigation on this matter as participants perceived access problems to be the most hindering factor for remission.

## Competing interests

The authors declare that they have no competing interests.

## Authors' contributions

CS designed the study, carried out the data collection, analyzed the data, and drafted the manuscript. SB analysed the qualitative data and assisted in writing the paper. AW conceived the study, participated in its design and coordination and helped to draft the manuscript. KL supervised the qualitative aspects of the study, was an auditor of the qualitative analysis and assisted in writing the paper. All authors read and approved the final manuscript.

## Pre-publication history

The pre-publication history for this paper can be accessed here:


